# Amiodarone promotes cancer cell death through elevated truncated SRSF3 and downregulation of miR-224

**DOI:** 10.18632/oncotarget.24385

**Published:** 2018-02-03

**Authors:** Yung-Lung Chang, Shu-Ting Liu, Yi-Wen Wang, Wei-Shiang Lin, Shih-Ming Huang

**Affiliations:** ^1^ Department of Biochemistry, National Defense Medical Center, Taipei City, Taiwan 114, Republic of China; ^2^ Department of Biology and Anatomy, National Defense Medical Center, Taipei City, Taiwan 114, Republic of China; ^3^ Division of Cardiology, Department of Medicine, Tri-Service General Hospital, National Defense Medical Center, Taipei City, Taiwan 114, Republic of China

**Keywords:** amiodarone, digoxin, caffeine, autophagy, miR-224

## Abstract

Amiodarone is a widely used class III antiarrhythmic agent which prolongs the action potential and refractory period by blockage of several types of myocardial potassium channels. Emerging evidence suggests that amiodarone sensitize tumor cells in response to chemotherapy. Nevertheless, little is known about the underlying molecular mechanism. To gain further insight, we demonstrated that amiodarone accumulated the population of a premature termination codon-containing isoform of serine and arginine rich splicing factor 3 (*SRSF3-PTC*) without increasing alternative spliced *p53 beta* isoform. Amiodarone enhanced reactive oxygen species production and increased cell apoptosis, whereas reduced DNA damage. Moreover, amiodarone suppressed miR-224 and increased its target *COX-2* expression. Taken together, our results suggested amiodarone caused cancer cell death might be through increased *SRSF3-PTC* and miR-224 reduction in a p53-independent manner.

## INTRODUCTION

Amiodarone is an anti-arrhythmic drug that is widely used to treat the most prevalent type of arrhythmia and atrial fibrillation [1–4]. It exerts its phamacologcal function through inhibition of diverse ion channels, including sodium, potassium, and calcium channels. In addition to antiarrhythmic activity, it has also been reported to possess anti-inflammatory and anti-oxidative properties [5, 6]. Amiodarone alters the function of diverse membrane proteins at therapeutic concentration, resulting in complex therapeutic and toxicity profiles [7, 8]. A treatment cocktail containing amiodarone reveals the promise for the treatment of prostate carcinomas and un-resectable hepatocellular carcinomas [9, 10]. However, the detailed mechanism by which amiodarone-induced cancer cell death remains unclear.

Amiodarone has been identified to be involved in the promotion of long-lived protein degradation in three autophagy-induced screening assays [11–13]. In addition, the degradation of autophagy-mediated micro RNA (miR)-224 is confirmed by the autophagy inducer amiodarone [14]. The overexpression of miR-224 promotes cell proliferation, anti-apoptosis, migration, and invasion via directly targeting the *TNFα-induced protein 1* (*TNFAIP1*), *SMAD4*, *caspase 3*, and *p21* in non-small cell lung cancer (NSCLC), colorectal cancer (CRC), and hepatocellular and prostate cancers [14–19]. Hence, amiodarone has the potential to be developed as an anti-tumor drug via the autophagy-mediated miR-224 degradation or other pathways.

The importance of mRNA splicing is highlighted for tissue homeostasis and disease progression. Alternative splicing might affect all areas of tumor biology, including metabolism, cell cycle control, apoptosis, senescence, and epithelial-mesenchymal transition [20–23]. Upgraded studies have suggested that the human serine and arginine rich splicing factor 3 (*SRSF3*) gene generates two alternative spliced transcripts: 1. a major mRNA isoform (SRSF3*-FL*) encoding functional full-length protein; and 2. a premature termination codon (PTC)-containing isoform (SRSF3*-PTC*), which could be degraded through nonsense-mediated mRNA decay (NMD), i.e. a surveillance mechanism that decomposes PTC-containing mRNAs [24]. Some chemicals, such as caffeine, digoxin, and theophylline, facilitate the translation of a truncated SRSF3 (SRSF3-TC) protein transcribed from *SRSF3-PTC* mRNA [25–27]. This SRSF3-TC protein is different from full-length SRSF3, which is involved in stressful conditions, such as senescence, hepatocyte differentiation, and metabolic functions [22, 23, 28]. Knockout studies have indicated that SRSF3 is essential for mouse development, hepatocyte differentiation, and metabolic function, as well as tumor cell proliferation and maintenance [23, 28–30]. Cardiac glycosides may inhibit the NMD activity by the elevation of intracellular calcium levels mediated through the binding and inhibiting the sodium-potassium ATPase on the plasma membrane [31].

MiRNAs, a group of small non-coding RNAs, bind to their respective mRNA targets and mediate gene silencing to regulate a range of developmental and physiological processes via the RNA interference mechanism [32, 33]. The dysregulation of miRNAs has been associated with cancers via the role of oncogene or tumor suppressor, depending on the cellular context and the genes targeted. Recently, miRNAs have emerged as promising therapeutic targets mediated through their extraordinary regulatory potential to regulate entire signaling networks within the cells.

Alternative splicing and the RNA interference mechanism have the plasticity to remodel the proteome and, subsequently, to subvert the process to produce proteins for cancer cells to suit the needs of growing and spreading tumors. Recent studies, mechanistically, demonstrated that SLU7 modulates the splicing and expression of *SRSF3* and *HNF4α* genes, which are essential for the preservation of the hepatocyte identity [34]; amiodarone induces the autophagy-preferential degradation of miR-224 in hepatocellular carcinoma tumorigenesis [16]; increased miR-224, directly targeting the *TNFAIP1* and *SMAD4* expression, functions as a potent oncogenic miRNA to promote cell migration, invasion, and proliferation in NSCLC [18, 19]. We further examined the potential working mechanism of amiodarone via splicing factors and miRs for its anti-tumorigenicity. Our current work and the literature may provide a repurposing function for amiodarone in clinical applications.

## RESULTS

### Amiodarone downregulated SLU7 and SRSF3 splicing factors in HeLa cells

**Figure 1:**
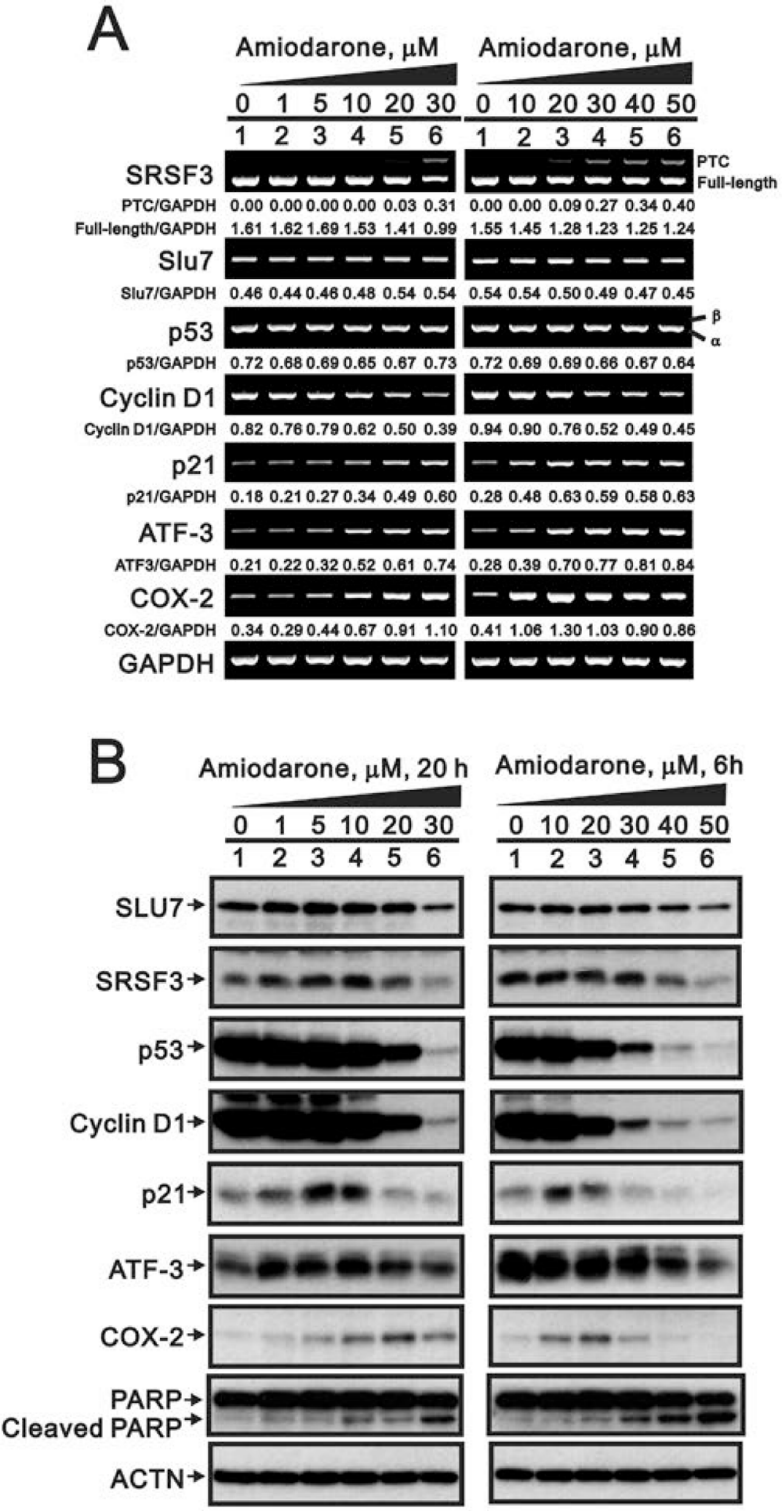
The effects of amiodarone on target gene and protein expression in HeLa cells HeLa cells were treated with indicated amount of amiodarone for indicated time. The cells were collected and subjected to (**A**) RT-PCR analysis of *SRSF3*, *Slu7*, *p53*, *cyclin D1*, *p21*, *ATF3*, *COX-2*, and *GAPDH* (loading control) mRNA expression; (**B**) immunoblot analysis for the detection of SLU7, SRSF3, p53, cyclin D1, p21, ATF3, COX-2, PARP, and ACTN (loading control) protein expression. PCR bands (A) were quantified through pixel density scanning and evaluated by ImageJ software, version 1.44a (http://imagej.nih.gov/ij/). The results are representative of two independent experiments.

Recent studies have suggested that amiodarone might have similar effects as caffeine or digoxin on the SRSF3-p53 pathway for senescence functions [7, 8, 22, 23]. Hence, we examined the effects of amiodarone on the alternative splicing of *SRSF3* and *p53*. Our data demonstrated that *SRSF3-PTC*, the alternative-spliced *SFSF3*, was observed at the high dose (30 µM) of amiodarone treatment, whereas no alternative splicing *p53β* form was detected in HeLa cells (Figure 1A). A precious study suggests that Slu7 is responsible for *SRSF3* normal transcription [34]. We consistently observed the downregulation of *Slu7* mRNA expression by amiodarone in a dose-dependent manner. For SLU7 is also known as a negative regulator of *ATF3* mRNA expression, we observed amiodarone dose-dependently enhanced *ATF3* mRNA expression in HeLa cells. In addition, *cyclin D1* mRNA, as well as *Slu7* mRNA, was reduced and *p21*, *COX-2* mRNAs, as well as *ATF3* mRNA were induced in an amiodarone dose-dependent manner.

In the Western blotting analysis, we observed that the decreasing pattern of SLU7, SRSF3, p53, and cyclin D1 proteins followed by amiodarone treatment in a dose-dependent manner in HeLa cells (Figure 1B). COX-2 and p21 proteins were induced at the narrow dosage window. In contrast to mRNA expression pattern, ATF3 proteins were reduced by amiodarone in a dose-dependent manner in HeLa cells. We also observed amiodarone induced the protein levels of cleaved-PARP in a dose-dependent manner, suggesting that amiodarone might induce apoptosis in HeLa cells.

### Amiodarone induced cell death and suppressed cell survival in HeLa cells

**Figure 2:**
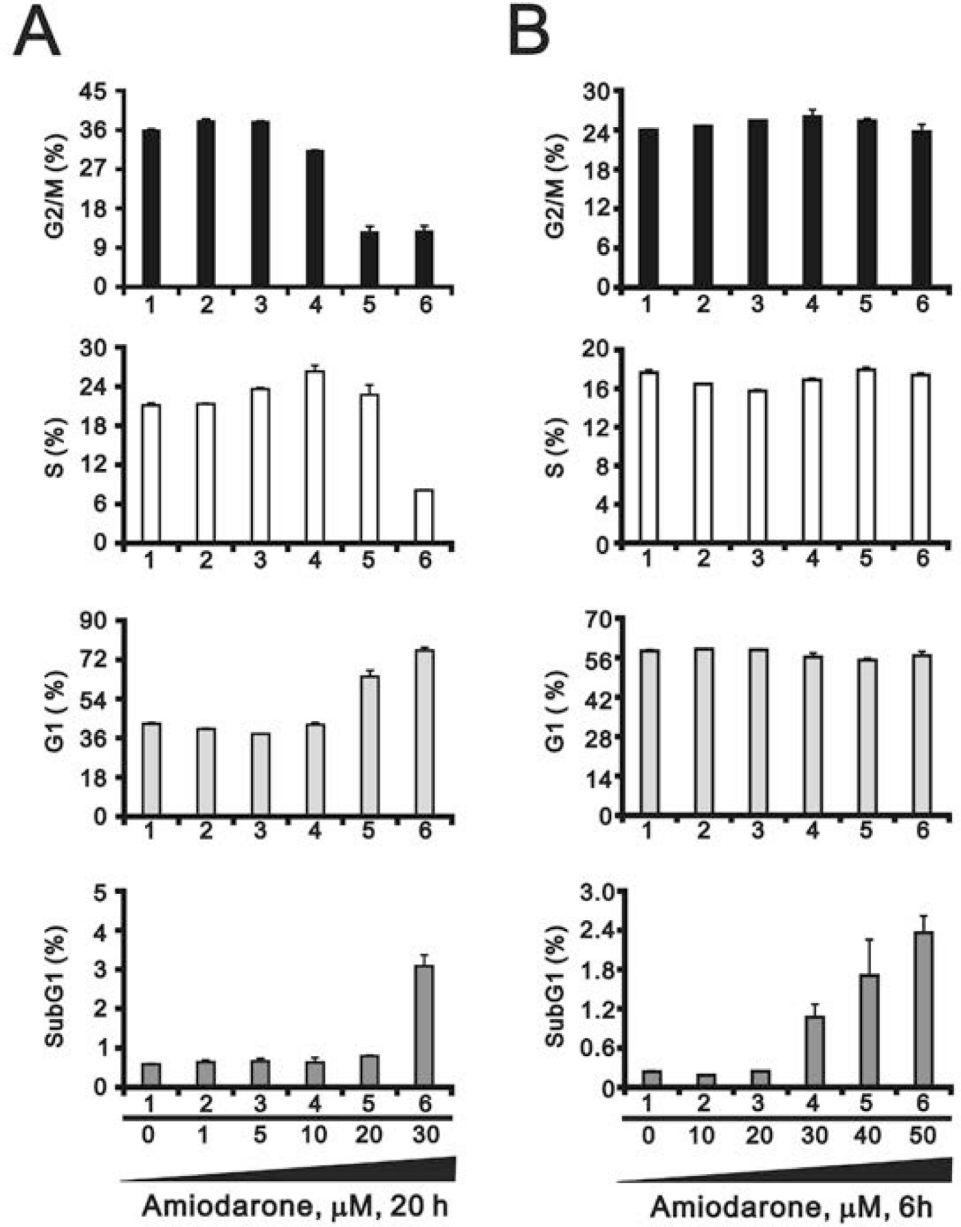
Amiodarone caused cellular apoptosis HeLa cells were treated with (**A**) lower concentration of amiodarone for 20 h and (**B**) higher concentration of amiodarone for 6 h. The cells were stained with PI, and the DNA content was determined by FACS analysis. The results are representative of three independent experiments.

**Figure 3:**
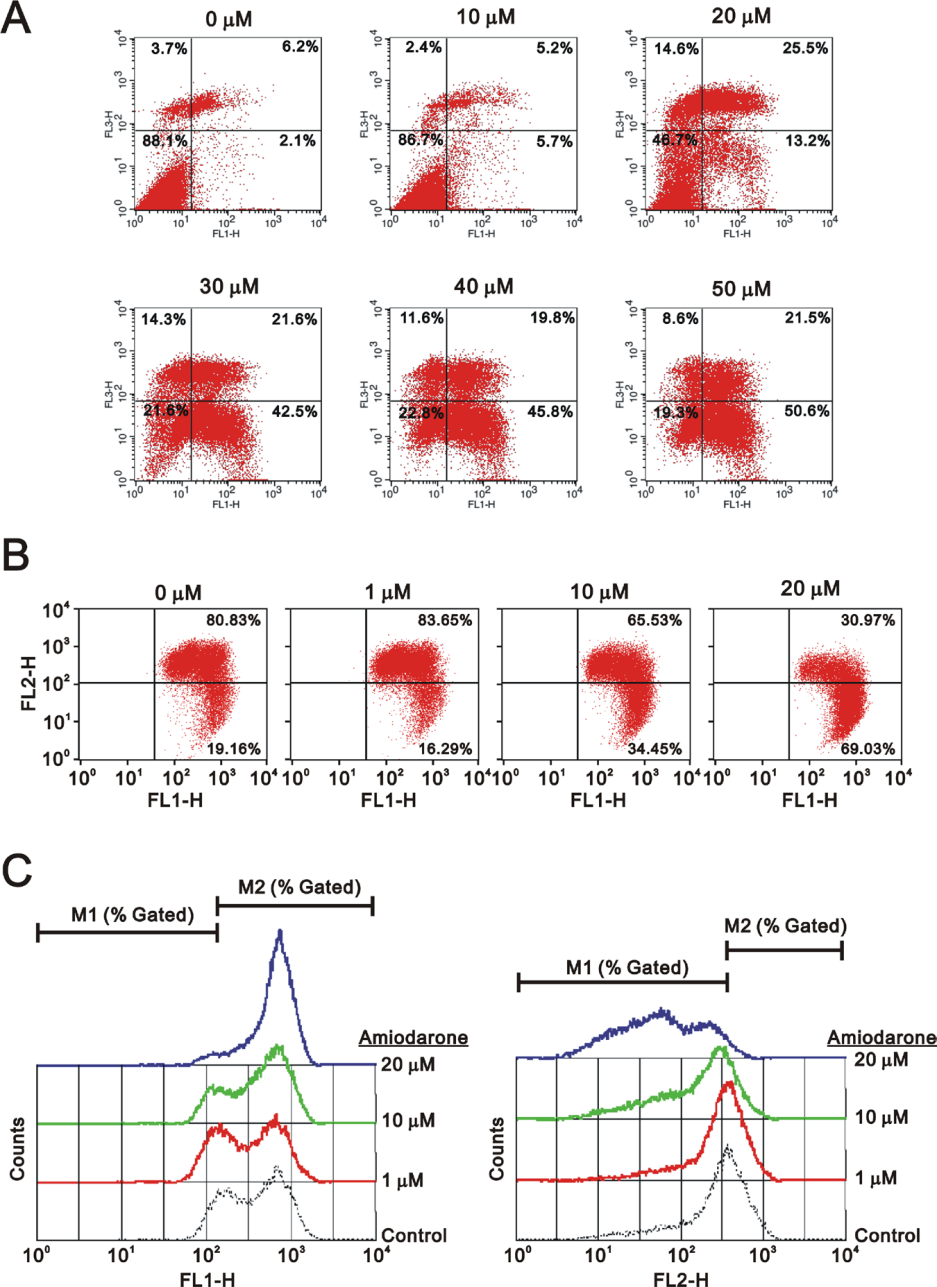
Amiodarone triggered intrinsic apoptosis HeLa cells were treated indicated amount amiodarone for 20 h. Cells were subject to the flow cytometry analysis using (**A**) the PE Annexin V Apoptosis Detection Kit (BD Biosciences) (**B**) the JC-1 staining kit. The results (A and B) are representative of three independent experiments. (**C**) The data from (B) were further measured the values of FL1-H and FL2-H.

**Figure 4:**
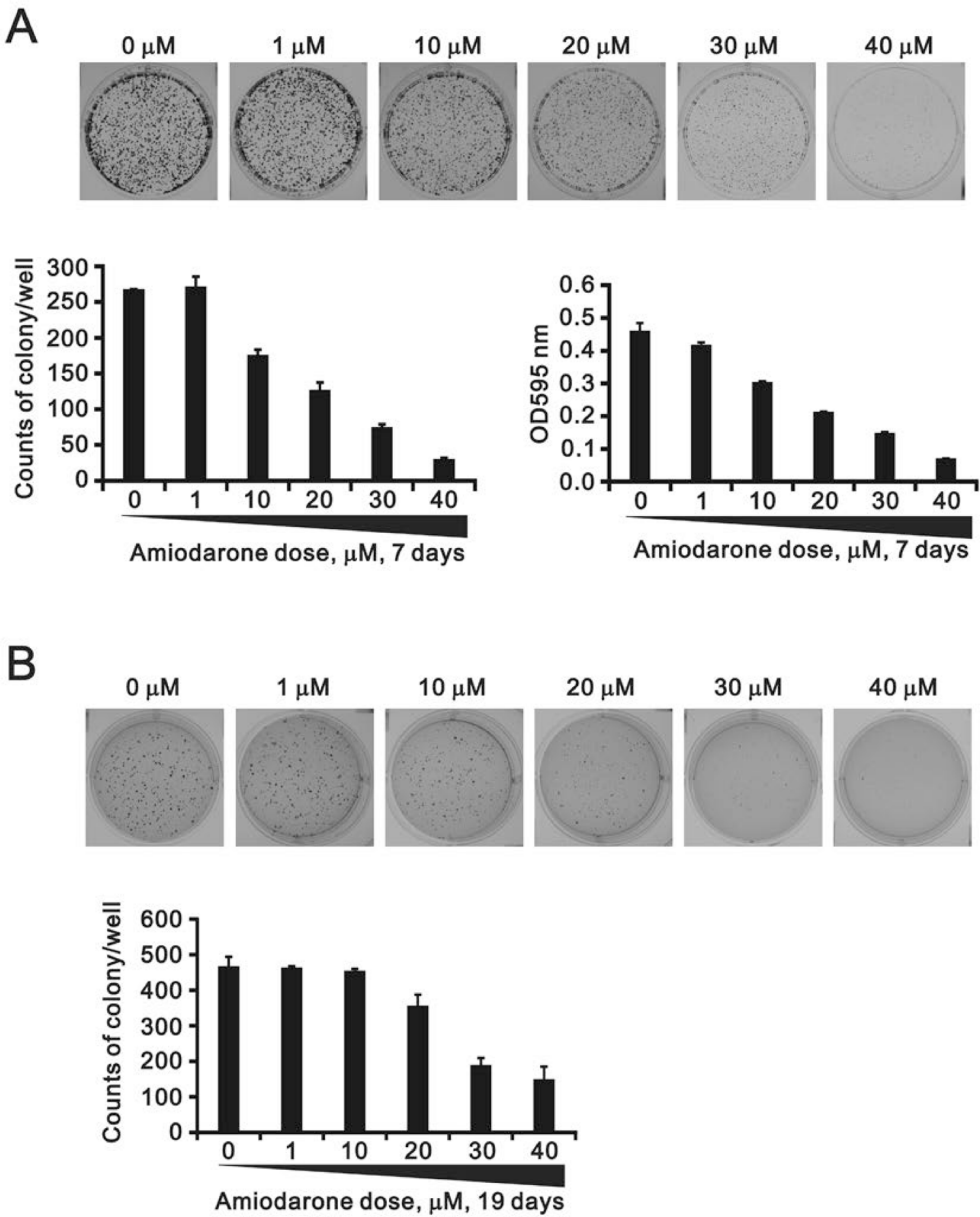
The effects of amiodarone on the cell colony and anchorage-independent growth (**A**) HeLa cells were treated indicated amount amiodarone for 7 days and cells were subject to the colony formation. After 7 days incubation, colonies were fixed with methanol and stained with 0.005% crystal violet solution for 1 h and photographed. Colonies were counted and quantified using ImageJ software. (**B**) HeLa cells were treated indicated amount amiodarone for 19 days and cells were subject to the colony formation in soft agar. After 19 days incubation, colonies were stained with 0.005% Crystal Violet solution for 30∼60 min and photographed. Colonies were counted and quantified using ImageJ software. All assays were done in triplicate.

To determine the effects of amiodarone on cell cycle profile, we performed cell cycle analysis using flow cytometry. Amiodarone treatment for 20 hours significantly increased populations at sub-G1 and G1 phase (G1 arrest) accompanied by the decreasing populations at S phase and G2/M phase (Figure 2A). However, there was no apparent effect, except for the sub-G1 phase, in the 6 h amiodarone treatment (Figure 2B). We further analyzed the amiodarone-induced cell death, including early- and late-apoptosis and necrosis using the Annexin V apoptosis kit. We observed the maximal percentages of late-apoptosis and necrosis at 20 µM amiodarone. The maximal percentage of early apoptosis was found at 50 µM amiodarone (Figure 3A). We examined the effect of amiodarone on the mitochondrial membrane potential using the JC-1 staining assay (Figure 3B). Our data suggest that amiodarone strongly disrupted the mitochondrial membrane potential via the analysis of FL1-H (monomers) and FL2-H (aggregates), which shows a trend to induce apoptosis in HeLa cells (Figure 3C and Table 1). To determine the effects of amiodarone on the cell survival and anchorage-independent ability, we evaluated the effects of amiodarone on HeLa cells using colony formation analysis and soft agar assay, respectively. We observed that amiodarone inhibited colony formation (Figure 4A) and decreased the anchorage-independent growth of HeLa cells (Figure 4B).

**Table 1:** The analysis of FL1 and FL2 shift trends from the JC-1 staining assay

amiodarone µM	FL1-H		FL2-H	
	M1 (% gated)	M2 (% gated)	M1 (% gated)	M2 (% gated)
**20**	5.33	94.73	96.05	4.09
**10**	15.68	84.52	81.02	19.39
**1**	23.04	77.33	62.10	38.55
**0**	14.80	85.57	61.05	39.40
	**right shift**		**left shift**	

### Amiodarone reduced SRSF3 gene and protein expression in HeLa and glioma cells

**Figure 5:**
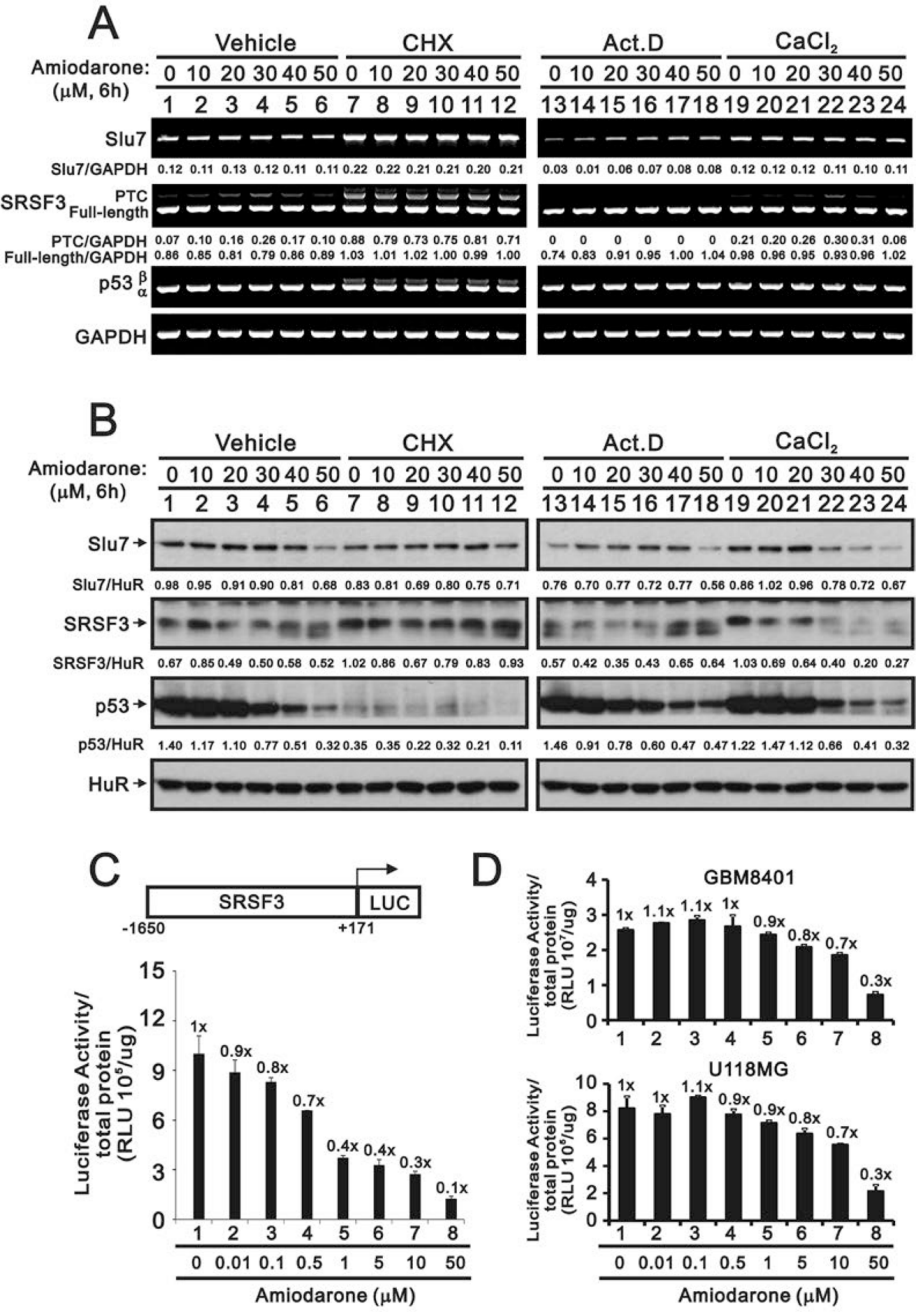
The regulatory mechanism of amiodarone on the SRSF3 expression HeLa cells were treated indicated amount amiodarone with pre-treated 2 h of vehicle, CHX (50 mg/ml), Act D (1 µM), and calcium chloride (1 mM) for 6 h. Cells were subject to the (**A**) Western blotting analysis and (**B**) RT-PCR analysis. The numerical data below each band is to indicate the ratio of specific mRNA (A) or protein (B) with control mRNA (*GAPDH*) or control protein (HuR) using the ImageJ software. The results (A and B) are representative of three independent experiments. (**C** and **D**) HeLa, GBM8401, U118MG cells were transiently transfected with 0.5 mg pGL3.SRSF3 (–1650/+171)-LUC and cells were treated with indicated amount of amiodarone for 20 h. Cells were harvested for luciferase reporter assay with the Promega Luciferase Assay Kit. The numbers (C and D) above the columns indicate the luciferase activity relative to an index of 1 for the reporter alone with vehicle.

As previous study and Figure 1 data suggest that SLU7 is responsible for *SRSF3* normal transcription and the downregulation of *Slu7* mRNA expression by amiodarone in a dose-dependent manner [34], we further examined the relationship between *Slu7* and *SRSF3* at the translation and transcription levels by the treatment of respective inhibitor, cycloheximide (CHX) and actinomycin D (Act D). CHX increased the expression of the *Slu7* mRNA and subsequently elevated the levels of truncated and full-length *SRSF3* mRNAs and *p53β* mRNA in HeLa cells (Figure 5A, compare lanes 7–12 with lanes 1–6). From the aspect of protein half-life, our data were consistent with previous literature in which indicate p53 is a labile protein, whereas SLU7 and SRSF3 are stable in the presence of CHX (Figure 5B, compare lane 7 with lane 1). The expression trend of SLU7 protein was consistent with its mRNA expression in HeLa cells. The protein levels of full-length SRSF3 were positively correlated with the expression of SLU7 protein in HeLa cells (Figure 5B). Regarding the effects of amiodarone at transcrioptional levels, we observed the instability of *Slu7* mRNA in Act D-treated HeLa cells (Figure 5A, compare lane 13 with lane 1), and amiodarone dose-dependantly enhanced the stability of *Slu7* mRNA. We also found no truncated *SRSF3* mRNA was transcribed by actinomycin D implying that the induction of SRSF3-PTC by amiodarone was derived from the newly synthesized mRNA(Figure 5A, lane 13–18). The effects of amiodarone on *SRSF3-PTC* induction were enhanced with the addition of calcium chloride (Figure 5A, compare lanes 19–24 with lanes 1–6). The repressive effects of amiodarone on the SLU7 and SRSF3 proteins were potentiated with the addition of calcium chloride treatment (Figure 5B, compare lanes 19–24 with lanes 1–6).

We further examined the effect of amiodarone on the *SRSF3* promoter activity using the *SRSF3*(–1650/+171)-LUC reporter system. We observed the suppressive effect on the *SRSF3* promoter activity by amiodarone in a dose-dependent manner (Figure 5C). The suppressive effects of amiodarone on the *SRSF3* promoter activity were also verified in two glioma cell lines, GBM8401 and U118MG (Figure 5D).

### Amiodarone enhanced the efficacy of caffeine and digoxin on cytotoxicity in HeLa cells

**Figure 6:**
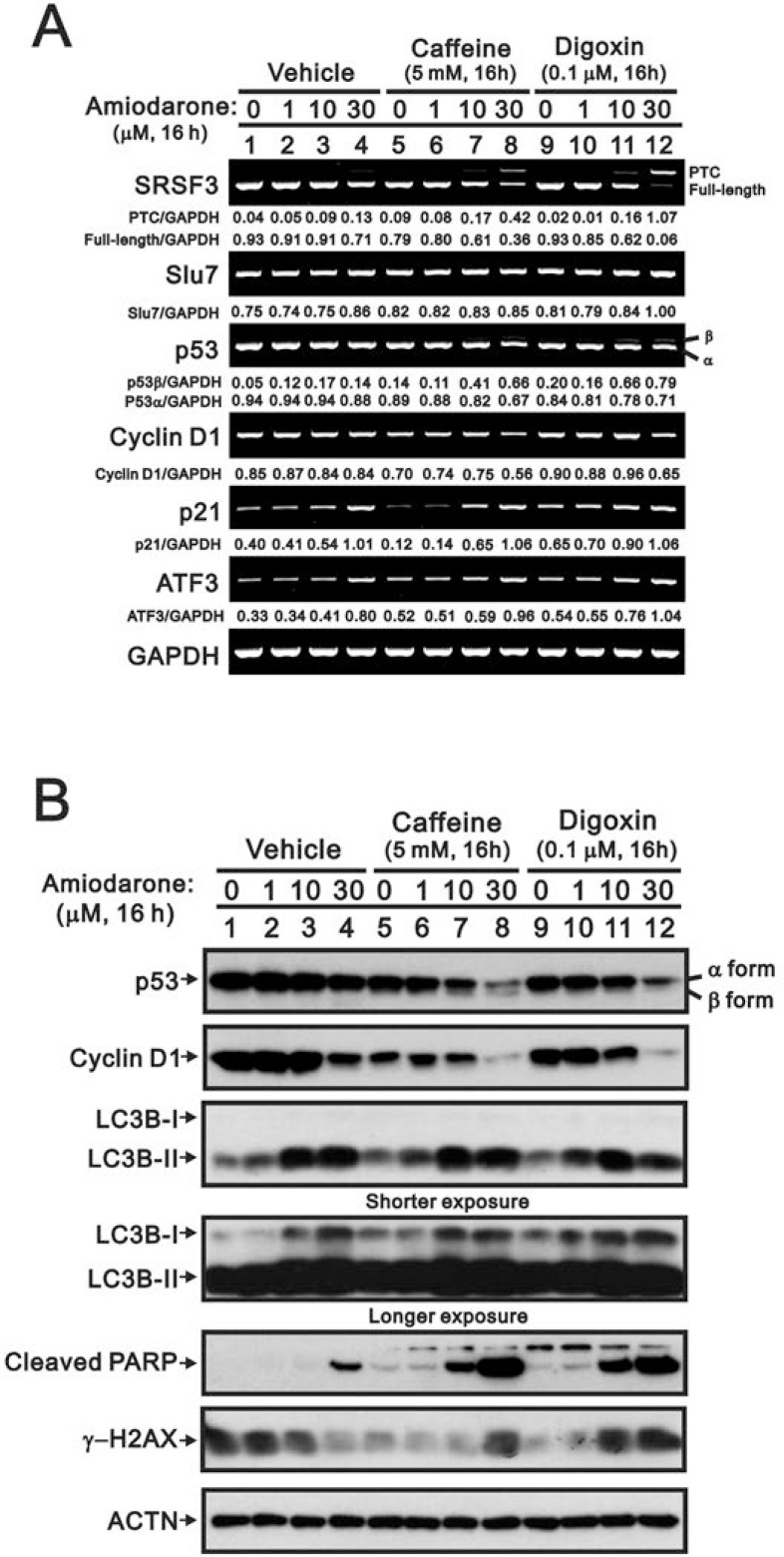
Amiodarone synergistically enhanced caffeine- or digoxin-induced SRSF3 alternative splicing and p53-independent apoptosis in HeLa cells HeLa cells were treated with 5 mM caffeine or 0.1 µM digoxin and along with indicated amount of amiodarone for 16 h. The cells were collected and subjected to (**A**) RT-PCR analysis of *SRSF3*, *Slu7*, *p53*, *cyclin D1*, *p21*, *ATF3*, *COX-2*, and *GAPDH* (loading control) mRNA expression; (**B**) immunoblot analysis for the detection of p53, cyclin D1, LC3B, PARP, γ−H2AX, and ACTN (loading control) protein expression. PCR bands (A) were quantified through pixel density scanning and evaluated by ImageJ software, version 1.44a (http://imagej.nih.gov/ij/). The results are representative of two independent experiments.

**Figure 7:**
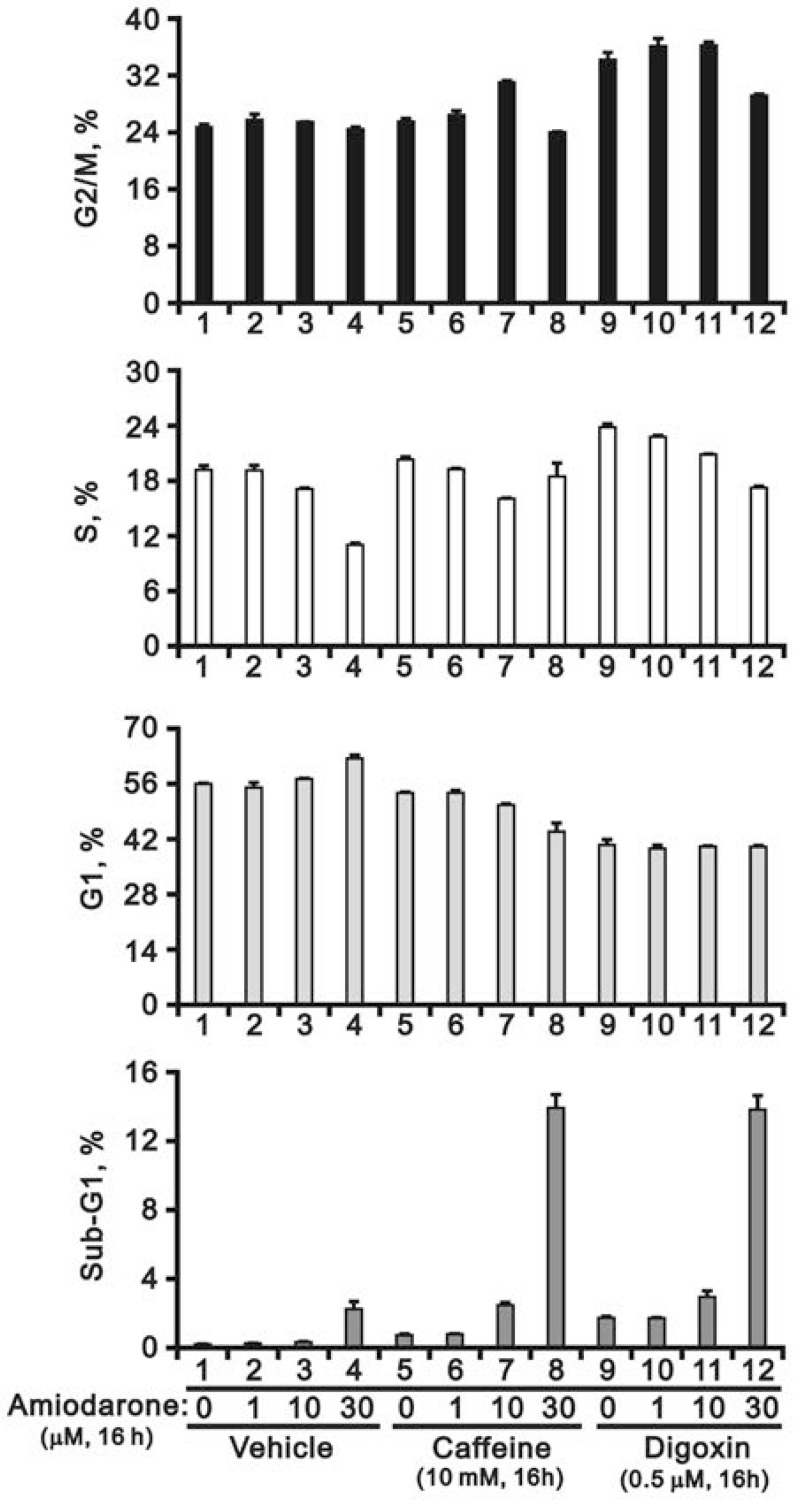
Amiodarone augmented the effects on the increase of sub-G1 population in HeLa cells by caffeine or digoxin HeLa cells were treated with 5 mM caffeine or 0.1 µM digoxin and along with indicated amount of amiodarone for 16 h. The cells were stained with PI, and the DNA content was determined by FACS analysis. The results are representative of three independent experiments.

Since our previous studies showed that caffeine and digoxin caused cell death via the SRSF3-p53 pathway [26, 27], we further examined the combined effects of amiodarone with caffeine and digoxin in HeLa cells. We found that amiodarone enhanced the alternative splicing effects on *SRSF3 (FL and PTC)* and *p53 (α and β)* by caffeine and digoxin, and in turn increased the mRNA levels of p53 target genes, such as *cyclin D1*, *p21*, and *ATF3* (Figure 6A). In the Western blotting analysis, amiodarone enhanced the degradation of p53 α and cyclin D1 proteins in HeLa cells (Figure 6B). Caffeine and digoxin enhanced the LC3B II levels (autophagy marker) and the cleaved PARP proteins (apoptosis marker) in the context of amiodarone treatment. In addition, amiodarone mitigated cellular DNA damage by monitoring the abundance of γ-H2AX in HeLa cells, whereas caffeine and digoxin reversed the dose-dependent alleviative effect of amiodarone on the DNA damage. Remarkably, Caffeine and digoxin synergistically enhanced the amiodarone-induced sub-G1 populations in cell cycle analysis using flow cytometry. We also found amiodarone alone or cotreated with either caffeine or digoxin, restrained the cell cycle from entering into S phase (Figure 7).

### Amiodarone induced cellular reactive oxygen species (ROS) and can be synergistically enhanced by caffeine or digoxin

**Figure 8:**
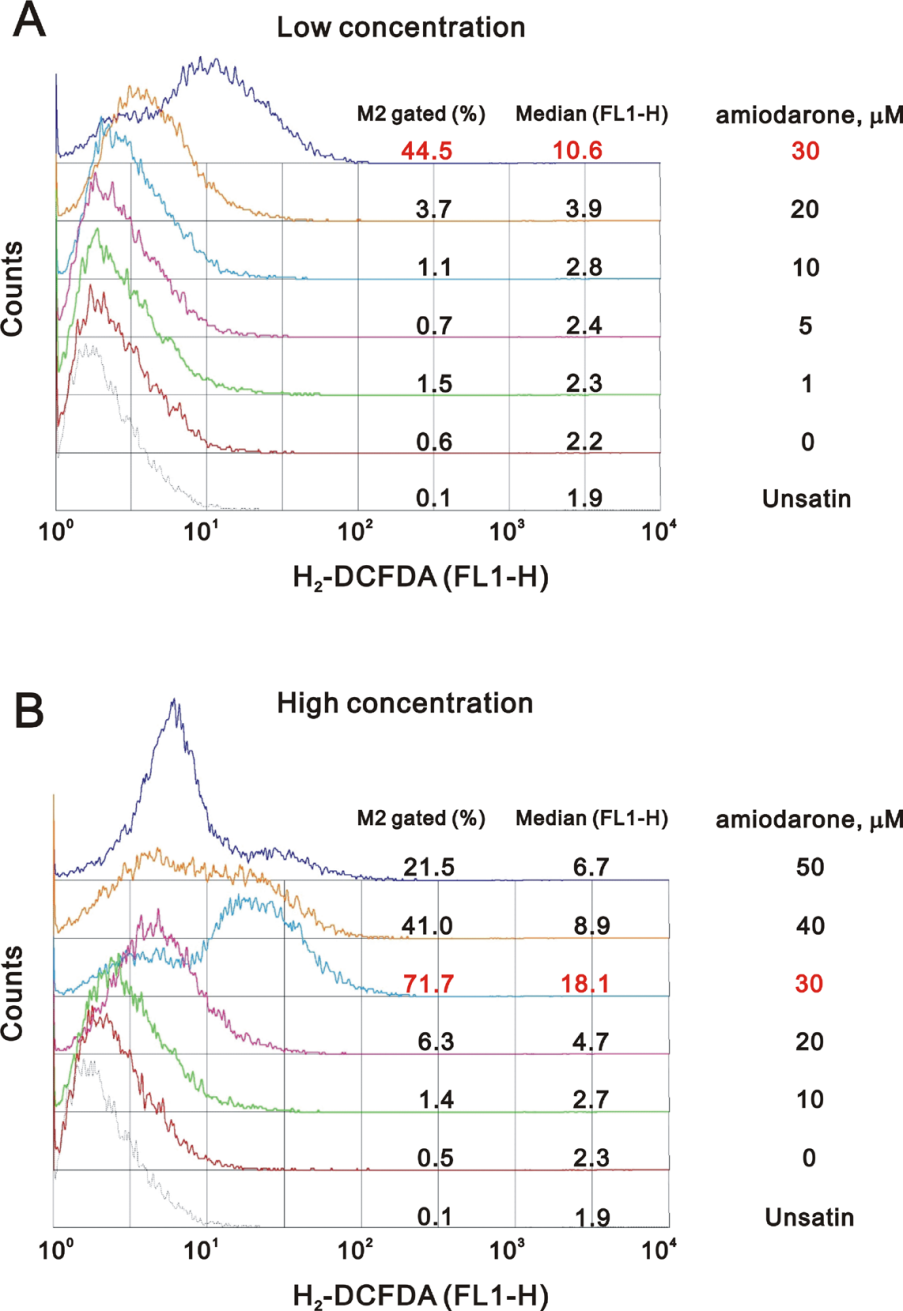
Amiodarone induced the ROS generation in HeLa cell HeLa cells were treated with (**A**) lower concentration of amiodarone for 20 h and (**B**) higher concentration of amiodarone for 16 h. After incubated, we stained live cells with 10 µM DCFH-DA for 30∼60 min at 37° C, harvested cells were then subjected to FACS analysis. The results are representative of three independent experiments.

**Figure 9:**
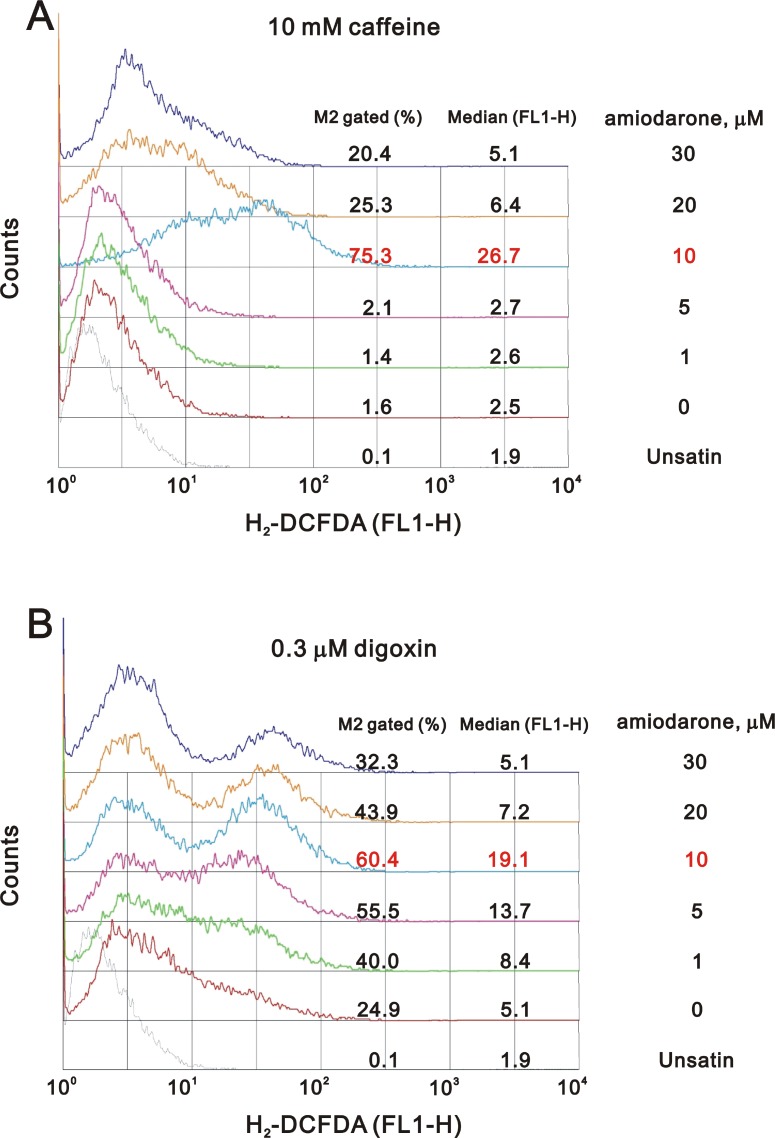
Caffeine or digoxin increased the effects of amiodarone for the elevation of ROS generation in HeLa cell HeLa cells were treated with (**A**) 10 mM caffeine or (**B**) 0.3 µM digoxin and lower concentration of amiodarone for 16 h. After incubation, we stained live cells with 10 µM DCFH-DA for 30∼60 min at 37° C, harvested cells were then subjected to FACS analysis. The results are representative of three independent experiments.

One of the underlying mechanisms by which amiodarone-induced pulmonary toxicity is the generation of ROS [35]. We further examined the status of ROS in the amiodarone treated HeLa cells. Our data demonstrated that the ROS in HeLa cells reached maximal amounts at 30 µM amiodarone treatment (Figure 8). Intriguingly, we found that caffeine and digoxin reduced the amiodarone concentration from 30 µM to 10 µM for the maximal amounts of ROS production (Figure 9). It is noteworthy that digoxin, not caffeine, enhanced the ROS generation in HeLa cells without amiodarone treatment.

### Amiodarone reduced miR-224 and other miR expressions in HeLa cells

**Figure 10:**
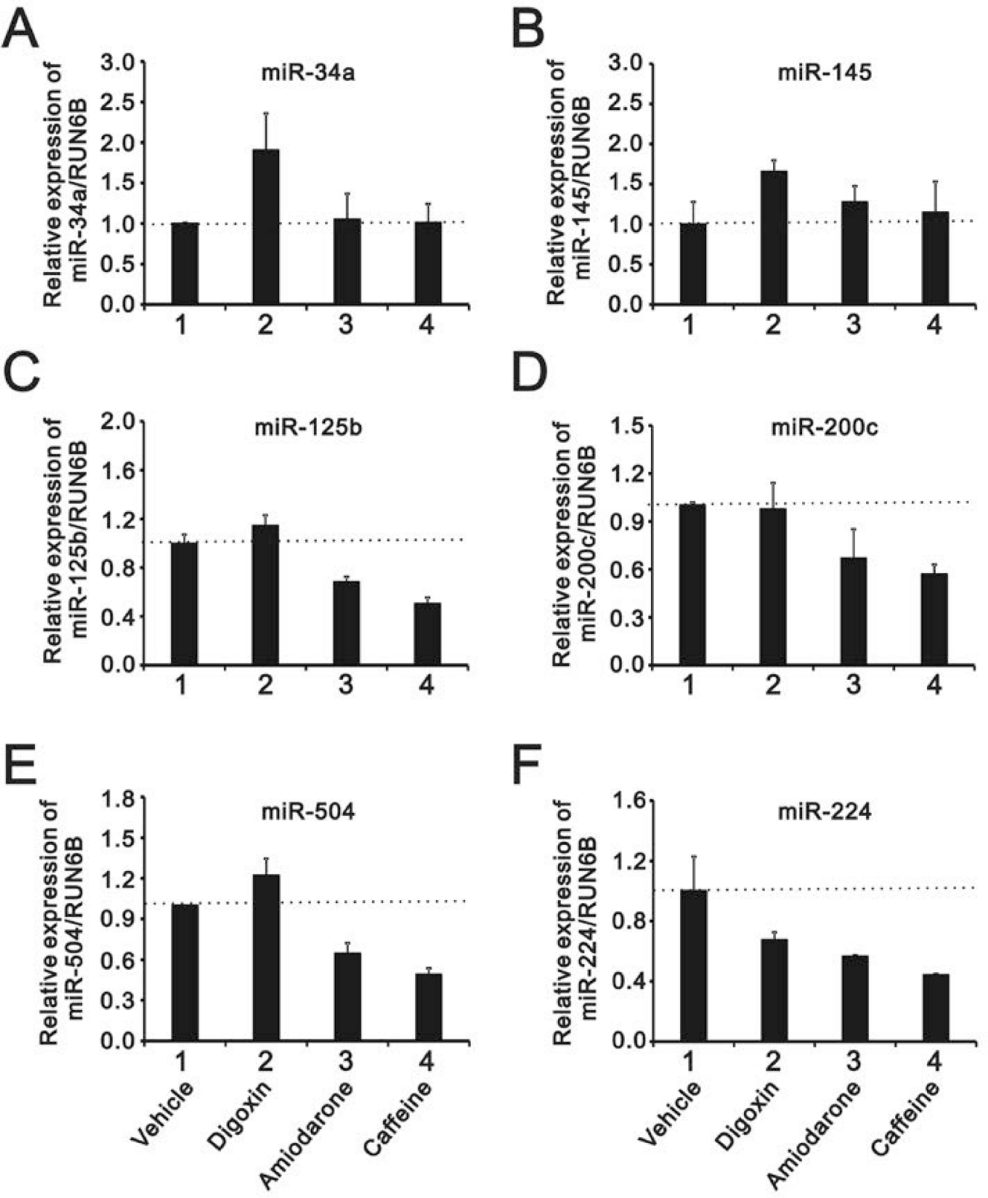
The comparison of the effects of caffeine, amiodarone, and digoxin on the miRs regulation in HeLa cell HeLa cells were treated with 10 mM caffeine, 0.3 µM digoxin, and 30 mM amiodarone for 16 h. The cells were collected and subjected to the miR analysis for (**A**) miR-34a; (**B**) miR-145; (**C**) miR-125b; (**D**) miR-200c; (**E**) miR-504; and (**F**) miR-224 expression. The results are representative of three independent experiments.

A recent study suggested that amiodarone-induced autophagy reduced the miR-224 expression at the post-transcriptional level [16], and our data supported that amiodarone did induce autophagy in HeLa cells (Figure 6B). Hence, we analyzed several miRs responsible for p53 proteins, including miR-34a, 145, 125b, 200c, and 504. In addition to miR-224, amiodarone also reduced the expression levels of miR-125b, 200c, and 504 in HeLa cells (Figure 10A–10F). Caffeine exhibited similar effects of abovementioned miRs downregulation with amiodarone (Figure 10C–10F), whereas digoxin showed opposite effects on enhanced miR-34a, 145, and 504 expressions in HeLa cells (Figure 10A, 10B and 10E).

**Figure 11:**
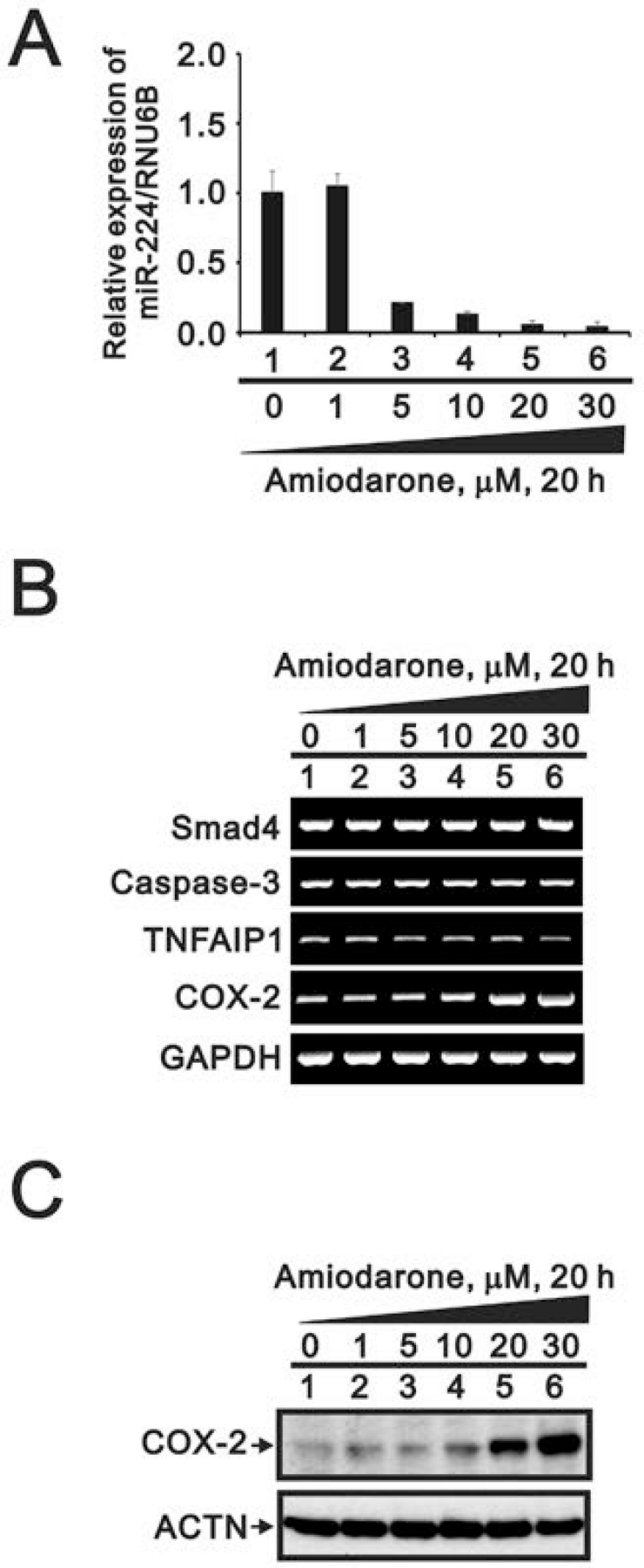
The effects of amiodarone on miR-224 and its target genes in HeLa cell HeLa cells were treated with lower concentration of amiodarone for 20 h. The cells were collected and subjected to (**A**) the miR analysis for miR-224 expression; (**B**) the RT-PCR analysis for *caspase3, TNFAIP1, COX-2,* and *GAPDH* (loading control); and (**C**) the Western blotting analysis using antibodies against COX-2 and ACTN (loading control). The results are representative of three independent experiments.

To further examined the effects of miR-224 reduction followed by amiodarone treatment, we detected miR-224 expression in response to various amiodarone concentration. We found that amiodarone repressed the miR-224 expression in a dose-dependent manner for 20 h (Figure 11A). However, we examined three miR-224 target genes: *Smad4*, *caspase 3*, and *TNFAIP1*; and found no apparent increasing effect accompanied by the amiodarone-reduced miR-224 (Figure 11B). We observed the *COX-2* gene and protein were increased by amiodarone in a dose-dependent manner. (Figure 11B and 11C). These results suggest that *COX-2* gene might be the target gene of miR-224 in response to amiodarone treatment in HeLa cells.

## DISCUSSION

The NMD pathway is well known as a translation-coupled quality control system [36–38]. Human *SRSF3* gene generates two alternative spliced transcripts, encoding full-length protein SRSF3-FL and a truncated SRSF3-TC isoform that is degraded through the NMD pathway [28]. Our current findings of the downregulation of *SRSF3-FL* expression and the upregulation of *SRSF3-PTC* expression were found by the treatment of caffeine, digoxin, and theophylline in HeLa and other cells [25–27]. Recent reporting system screening for hundreds of clinical drugs from Dr. You’s laboratory have demonstrated that caffeine (positive control in the screening system) and digoxin (the top drug in the listing) may elevate the intracellular cyclic AMP and calcium levels for the inhibitory effects on the NMD pathway [31]. Amiodarone was included in a positive category, but its screening ratio value was around 1 when compared to 30 for digoxin. We observed the *SRSF3-PTC* expression of at a higher amiodarone concentration, but it failed to alternatively splice *p53* from *α* isoform into *β* isoform via the downregulation of *SRSF3* gene and protein in our current observation window. However, amiodarone acted synergistically with caffeine and digoxin on the alternative splicing of *p53* from *α* isoform into *β* isoform via the downregulation of *SRSF3* gene and protein. Hence, our study revealed that amiodarone, as well as caffeine and digoxin, had common and distinctive working pathways in cells.

Amiodarone has been identified to be an autophagy inducer and autophagy-mediated miR-224 degradation was reconfirmed in this study. Up-regulated miR-224 was identified in several solid tumors including CRC, NSCLC, and hepatocellular carcinoma [14–19]. It subsequently suppressed its targets such as *TNFAIP1*, *p21*, *caspase 3* and *SMAD4*. MiR224 might regulate CRC growth by targeting *p21* to modulate CRC proliferation and prognosis. In addition, *p21* is a well-known p53 target gene [39]. Amiodarone might modulate the expression amount of *p21* via the miR-224 and p53 in HeLa cells. The downregulation of miR-224 and p53 by amiodarone has antagonistic effects on the *p21* mRNA via defined pathways. However, in some cases, *p21* could be regulated and mediated through p53-dependent and/or independent pathway(s). Our previous study demonstrated that the senescence concentration (0.1 µM) of doxorubicin (DXR) induced higher p21 expression than its apoptotic concentration (1 µM), at which the highest p53 abundance was measured in HeLa cells [40]. Correspondingly, we demonstrated that expression of p21 correlates with activation of p53 at low amiodarone concentration but not at high doses. Hence, amiodarone might reduce the miR-224 expression via the autophagy degradation pathway and subsequently to increase the p21 abundance followed by a p53-independent mechanism in our current work. In addition to the expression level in cells, cytoplasm localized p21 blocks the activation of pro-caspase 3 consequently inhibit apoptosis; whereas, the nuclear-localized p21 promotes its degradation and creates favorable conditions for the occurrence of apoptosis in response to DNA damage [41, 42]. Further investigation will be necessary to distinguish the subcellular localization of p21 after various DNA stress conditions.

Our previous study demonstrated that the senescence concentration (0.1 µM) of DXR induced higher percentage of DNA damage than its apoptotic concentration (1 µM) in HeLa cells, since senescence development is mainly controlled by the induction of p21 in cells associated with DNA damage (via the measurement of γ-H2AX) [40]. Amiodarone had the ability to reduce the DNA damage level and induce the autophagy and apoptosis levels, whereas caffeine or digoxin could reverse the amiodarone-reduced DNA damage level and potentiate the amiodarone-induced apoptosis levels. However, the differential levels of DNA damage responsible for cellular apoptosis and/or autophagy by amiodarone remain to be investigated in the future.

Previous studies have demonstrated that increased ROS levels contribute to enhanced tumor cell proliferation and apoptosis suppression, whereas high levels of ROS can also cause apoptosis via the release of pro-apoptotic factors from mitochondria [43, 44]. It is attractive to find targets which are responsible for enhancing mitochondrial ROS production to trigger apoptosis, because this organelle controls the decision for cells to live or die. In this study, we provided supporting data that Amiodarone can also modulate endogenous ROS levels in a dose-dependent manner, and disrupt the mitochondrial membrane potential to induce cellular intrinsic apoptosis. Hence, it may have clinical benefit to use amiodarone with other chemotherapeutic agents from the aspect of the increased ROS produced by triggering DNA damage response pathways or by altering the function of diverse membrane proteins via splicing factors.

An increasing number of studies have indicated that miRs play a critical role in malignant development via the inhibition of the mRNAs stability and/or translation, which can function as tumor suppressors or oncogenes in a tissue specific manner [45, 46]. MiR-224 has been reported to be up-regulated in CRC, hepatocellular, and prostate cancers [14–17]. Nevertheless, the regulation of downstream targets of miR-224 determines its physiological responses, especially affected by different agents A recent study of cervical cancer tissues has shown that up-regulated 195 miRNAs and down-regulated 96 miRs relative to normal cervical tissues [47]. We examined several well-known target genes of miR-224, such as *Smad4*, *caspase 3*, and *TNFAIP1* and found no change in amiodarone-reduced miR-224 cells in a human papillomavirus (HPV)-infected cervical carcinoma cell line, HeLa cells. *COX-2* was the only consistent induction of miR-224 target genes by amiodarone in this study. In addition to further investigate whether *COX-2* is a tissue-specific target gene of miR-224, the expression of *COX-2* mRNA is also induced by HPV type 16 E5, E6, and E7 oncoproteins and others [48–50]. Despite *COX-2* is also a target gene of p53 [51], inversion correlation shown in our results (Figure 1) indicated the induction of *COX-2* may be mediated via a p53-independent pathway in our model. The detailed regulatory mechanisms and functional roles of COX-2 in HPV type 18 HeLa cells remain to be investigated in the future.

Summarily, our data revealed that our interested gene expressions are modulated by amiodarone mediated through transcription, post-transcription, and/or translation. Multiple pathways of gene regulation of amiodarone provide a new avenue to define its repurposing functions alone or combined with other clinical drugs.

## MATERIALS AND METHODS

### Cell culture and chemicals

HeLa, GBM8401, U118MG cells were cultured in Dulbecco’s modified Eagle’s medium supplemented with 10% fetal bovine serum and 1% penicillin-streptomycin (Invitrogen, USA). Amiodarone, DCFH-DA, CHX, Act D, and calcium chloride were purchased from Sigma-Aldrich (A8423, D6883, C1988, A9415, and C1016, MO, USA).

### Western blot analysis

Cell lysates were prepared in lysis buffer (100 mM Tris-HCl of pH 8.0, 150 mM NaCl, 0.1% SDS, and 1% Triton X-100) at 4° C. The extracts were separated by SDS-PAGE, transferred onto a polyvinylidene difluoride membrane (Millipore, USA) and detected using antibodies against PARP, LC3 (Cell Signaling, USA), SRSF3, Slu7, p53, p21, cyclin D1, ATF3, COX-2, α-actinin (ACTN), HuR (Santa Cruz Biotechnology, USA), and γ−H2AX (Epitomics, USA).

### Reverse transcription-polymerase chain reaction (RT-PCR)

One microgram of total RNA, isolated using the TRIsure reagent (BIOLINE, UK), was subjected to the reverse transcription reaction using MMLV reverse transcriptase for 60 min at 37° C (Epicentre Biotechnologies, USA). The PCR reactions were run on a Veriti Thermal Cycler (Applied Biosystems, USA). Table 2 shows all PCR primer sequences.

**Table 2:** Primers were used in this study

Gene name	Primer sequence (5′→3′)
*p53*	Forward: 5-CTCTGACTGTACCACCATCCACTA-3′ Reverse: 5-GAGTTCCAAGGCCTCATTCAGCTC-3′
*SRSF3*	Forward: 5-ATGCATCGTGATTCCTGTCCATTG-3′ Reverse: 5-CTATTTCCTTTCATTTGACCTAGATC-3′
*cyclin D1*	Forward: 5-ATGGAACACCAGCTCC-3′ Reverse: 5-TCAGATGTCCACGTCCCGC-3′
*p21*	Forward: 5′-CTGAGCCGCGACTGTGATGCG-3′ Reverse: 5′-GGTCTGCCGCCGTTTTCGACC-3′
*ATF3*	Forward: 5′-GAGGATTTTGCTAACCTGAC-3′ Reverse: 5′-TAGCTCTGCAATGTTCCTTC-3′
*COX-2*	Forward: 5′-CTGAGCCGCGACTGTGATGCG-3′ Reverse: 5′-GGTCTGCCGCCGTTTTCGACC-3′
*Slu7*	Forward: 5-GATGGGAAGAGGGATCGGTGG-3′ Reverse: 5-CTGGAGGGGCATCCAAATGTTC-3′
*GAPDH*	Forward: 5-CTTCATTGACCTCAACTAC-3′ Reverse: 5-GCCATCCACAGTCTTCTG-3′
*Smad4*	Forward: 5′-GCATCGACAGAGACATACAGC-3′ Reverse: 5′-CAACAGTAACAATAGGGCAGC-3′
*TNFAIP1*	Forward: 5-GGACAAGAAGGACTCCTACCA-3′ Reverse: 5-CCTCCAACAAGGAGTTGTCG-3′
*Caspase 3*	Forward: 5-CTGGAATGACATCTCGGTCTG-3′ Reverse: 5-ACCAGGTGCTGTGGAGTATG-3′

### Fluorescence-activated cell sorting (FACS) analysis

FACS analysis was based on the measurement of DNA content for nuclei labeled with propidium iodide (PI). For cell cycle evaluation, cells were treated using procedures for the proliferation experiments, washed with ice-cold PBS, and incubated with PI solution (0.05% mg/ml in PBS, 0.1% Triton X-100, and 0.01% RNase) for 15 min at room temperature in the dark. Cells were then subjected to FACS and cell cycle analysis was performed using a FACSCalibur flow cytometer (BD Biosciences, USA). For early and late apoptosis analysis, the cells were treated and measured by PE Annexin V Apoptosis Detection Kit (BD Biosciences) according to the manufacturer’s instructions.

### Mitochondrial membrane potential measurements

The mitochondrial potential was performed using BD™ MitoScreen Flow Cytometry Mitochondrial Membrane Potential Detection Kit (BD Biosciences), according to the manufacturer’s instructions. In brief, HeLa cells were seeded on a 6 cm culture plate and incubated with the amiodarone dose. After 18 h treatment, cells were trypsinized and collected cell pellets at 1000 rpm, resuspended the cells in PBS and to count cell number, cell counting should not exceed 1 × 10^6^ cells per ml. All the treated cells were the stained with the JC-1 dye for 10∼15 min at 37° C in a CO_2_ incubator. After washing twice with 1x Assay Buffer, the pellets were resuspended in 1x Assay Buffer (0.5 ml) and the fluorescence of the JC-1 dye was measured by flow cytometric analysis by exciting the dye at 488 nm and detecting the JC-1 monomer through its emission at 530 nm (FL1 channel) with aggregates of JC-1 being detected at 580 nm (FL2 channel).

### Immortalization and transformation assays

For colony formation at low density to assess immortalization, a total of 3,000 HeLa cells were seeded per well (six-well plate) and treated with amiodarone dose. Subsequently, the culture plates were kept in culture incubator maintained at 37° C and 5% CO_2_ for 7 days to allow for colony growth. After 7 days incubation, colonies were fixed with methanol and stained with 0.005% crystal violet solution for 1 h and photographed. Colonies were counted and quantified using ImageJ software. For colony formation in soft agar, a total of 3,000 HeLa cells were resuspended in 10% FBS DMEM medium containing 0.35% low melting temperature agarose (SeaPlaque^®^ Agarose; Lonza) and seeded onto six-well plates previously coated with 0.5% low melting temperature agarose. After 19 days incubation, colonies were stained with 0.005% Crystal Violet solution for 30∼60 min. Colonies were counted and quantified using ImageJ software.

### *SRSF3* promoter reporter construction, transfection, and reporter assay

The reporter vector for *SRSF3* promoter from –1650 to +171 was constructed into the pGL3-LUC vector, pGL3.SRSF3(–1650/+171)-LUC, via the *Bam*HI/*Xho*I site of a PCR fragment. HeLa, GBM8401, U118MG cells were transfected into 24-well plates with jetPEI (Polyplus Transfection Inc., New York, NY) according to the manufacturer’s protocol. The total DNA was adjusted to 1.0 μg by the addition of the empty vector. The cells were treated with vehicle or indicated amount of amiodarone for 20 h and were harvested for luciferase reporter assay, which was performed with the Promega Luciferase Assay Kit; the values are expressed numerically as relative light units normalized by total proteins. The luciferase activities are presented as the mean ± SD of three transfected wells and are representative of at least three independent experiments.

### Reactive oxygen species (ROS) measurement

Intracellular ROS levels were determined using the fluorescent marker DCFH-DA according to the manufacturer’s instructions. The cells were treated with indicated drugs (amiodarone, caffeine, and/or digoxin) for 16 h and then live cells were stained with 10 µM DCFH-DA for 30∼60 min at 37° C. The harvested cells and washed twice with PBS and then were subjected to FACS. The 2′,7′-dichlorofluorescein (DCF) fluorescence intensity analysis was performed using a FACSCalibur flow cytometer and the Cell Quest Pro software (BD Biosciences, CA, USA).

### MiR extraction and quantitative polymerase chain reaction (qPCR)

Total RNA was extracted from growing cells using the TRIsure reagent (Bioline Reagents, London, UK) and the miRNA reverse transcription reactions were using miScript II RT kit (Qiagen) according to the manufacturer’s instructions. Expression of miRNA was quantified by qRT-PCR with miScript SYBR Green PCR Kit (Qiagen). Small endogenous nucleolar U6 snRNA was used as control for normalization of miRNA. Target miR primers were purchased from miScript Primer Assay (Qiagen).

## Abbreviations

ACTN: α-actinin
CRC: colorectal cancer
DCF: 2′,7′-dichlorofluorescein
DCFH-DA: 2′,7′-dichlorofluorescin diacetate
DXR: doxorubicin
FACS: fluorescence-activated cell sorting
miR: HPV: human papillomavirus; micro RNA
NMD: nonsense-mediated mRNA decay
NSCLC: non-small cell lung cancer
PI: propidium iodide
PTC: premature termination codon
qPCR: quantitative polymerase chain reaction
ROS: reactive oxygen species
RT-PCR: reverse transcription-polymerase chain reaction
SRSF3: serine and arginine rich splicing factor 3
SRSF3-TC: truncated SRSF3
TNFAIP1: TNFα-induced protein 1
